# Model Composites Based on Poly(lactic acid) and Bioactive Glass Fillers for Bone Regeneration

**DOI:** 10.3390/polym13172991

**Published:** 2021-09-03

**Authors:** Xavier Lacambra-Andreu, Nora Dergham, Marlin Magallanes-Perdomo, Sylvain Meille, Jérôme Chevalier, Jean-Marc Chenal, Abderrahim Maazouz, Khalid Lamnawar

**Affiliations:** 1 CNRS, UMR 5223, Ingénierie des Matériaux Polymères, INSA Lyon, Université de Lyon, F-69621 Villeurbanne, France; xavier.lacambra@insa-lyon.fr (X.L.-A.); nora.dergham@gmail.com (N.D.); abderrahim.maazouz@insa-lyon.fr (A.M.); 2 CNRS, UMR 5510, MATEIS, INSA-Lyon, Université de Lyon, F-69621 Villeurbanne, France; marlin.magallanes-perdomo@insa-lyon.fr (M.M.-P.); sylvain.meille@insa-lyon.fr (S.M.); jerome.chevalier@insa-lyon.fr (J.C.); 3 Hassan II Academy of Science and Technology, Rabat 10100, Morocco

**Keywords:** biomedical-grade composites, extrusion processing, injection molding, rheology, PLA, bioglass, mechanical properties

## Abstract

Poly(l-lactide-co-d,l-lactide) PDLA/45S5 Bioglass^®^ (BG) composites for medical devices were developed using an original approach based on a thermal treatment of BG prior to processing. The aim of the present work is to gain a fundamental understanding of the relationships between the morphology, processing conditions and final properties of these biomaterials. A rheological study was performed to evaluate and model the PDLA/BG degradation during processing. The filler contents, as well as their thermal treatments, were investigated. The degradation of PDLA was also investigated by Fourier transform infrared (FTIR) spectroscopy, size-exclusion chromatography (SEC) and mechanical characterization. The results highlight the value of thermally treating the BG in order to control the degradation of the polymer during the process. The present work provides a guideline for obtaining composites with a well-controlled particle dispersion, optimized mechanical properties and limited degradation of the PDLA matrix.

## 1. Introduction

Among the latest generation of biomedical grade composites, some combine inorganic fillers and a polyester matrix in order to obtain osteoinductive and resorbable materials [1]. These composites can present tunable mechanical properties depending on the nature and fraction of the inorganic and organic phases and their physicochemical interactions. In particular their Young’s modulus can be tailored to fit with those of cancellous [2] or cortical bone [3]. In addition to improve the mechanical and bioactive properties of the composite, calcium phosphates and bioactive glasses (the main fillers used in orthopedic applications) present the advantage of buffering the acidic degradation of the polyester matrix [4]. It is important to this phenomenon because a local accumulation of acidic products can induce an inflammatory response from host tissues [5,6].

Bioglasses (BG) are bioactive, osteoconductive and osteoinductive. They can be divided into three families: silicate (45S5 Bioglass^®^, 13-93), borate (13-93B3) and phosphate (CaP glass) [7]. In this study, 45S5 was chosen as a filler. 45S5 Bioglass^®^ (45% SiO2; 24.5% Na_2_O; 24.5% CaO; 6% P_2_O_5_) was developed by Hench et al. in 1971 [8]. After implantation, a carbonated hydroxyapatite (C-HA) layer is formed in the BG surface, followed by the attachment and proliferation of osteoblasts. The mechanism of bonding to bone has been previously described in the literature [7].

The combination of BG and a resorbable polymer makes it possible to meet the mechanical and physiological demands of the host tissue [4]. The bioactivity and non-cytotoxicity of these composites have been widely demonstrated by in vivo studies [9,10,11]. Thermal treatments of BG have been studied to control and increase the crystallization, resorption and mechanical properties of BG [10,12,13,14]. The authors demonstrated that the degree of crystallinity does not reduce the ability of the BG to form C-HA, even if the kinetics are slowed down as compared to untreated BG [9,15,16]. In the literature, poly(lactic-acid (PLA)/BG composites have been processed mainly through two different techniques: dissolution and extrusion.

In the dissolution technique, the polymeric matrix is dissolved in a solvent and BG particles are added to this solution under agitation. Afterwards, the solution is precipitated and the composite is dried and ground into pellets. These pellets can be transformed into implants using a thermomechanical process. This entire process is time consuming and rather inconvenient from an industrial viewpoint. The second technique involves melt blending at elevated temperatures using extrusion, followed by injection molding of the implants. Several degradation descriptors have been reported during the processing of composites by this second method, such as bubble formation and the coloration of composites [17,18]. Various authors have demonstrated that, in the presence of BG, the high-temperature processing of PLA can cause a significant reduction of its molar mass and mechanical properties [19,20]. Unfortunately, few research efforts have been dedicated to controlling the degradation that takes place during the processing of PLA and BG fillers, especially in the melt state. Despite the critical aspect of this issue, only a handful of studies (see for Blaker et al. [17,18,21] Simpson et al. [22], Conoscenti et al. [23] and Larrañaga et al. [20]) have attempted to understand the mechanisms governing the interaction between BG fillers and the PLA matrix in the melt state. As the specific surface area of the BG particles (their size) and a potential (partial) crystallization are known to influence their bioactivity and their dissolution rate, it is hypothesized that these factors would also play a role in the degradation rate of the PLA during processing.

The aim of the present work is to better understand the relationships between the morphological, rheological and mechanical properties and the processing conditions of such BG-PLA composites. Bioglasses of various sizes that were subjected to different thermal treatments were used as fillers for an amorphous PDLA matrix. As described below, a special extrusion-injection process with in-situ monitoring process been developed in this work.

## 2. Materials and Methods

### 2.1. Materials

A Poly(l-lactide-co-d,l-lactide) hereafter referred to as PDLA, with a 98 L/2 DL ratio, a weight-average molecular mass (${\;\bar{M}}_{w})$ of $105\;000\;g\cdot {\mathrm{mol}}^{-1}$, a number-average molecular mass (${\;\bar{M}}_{n})\;\mathrm{of}\;62,000\;g\cdot {\mathrm{mol}}^{-1}$, an inherent viscosity of $2\;\mathrm{dL}\cdot g^{-1}$ and a density of 1.24 g·cm^−3^ was supplied by Nature Works LLC (USA) (reference: PLA 4060D). Before the extrusion process, the PDLA was dried for 4 h at 45 °C under vacuum in order to prevent hydrolytic degradation during the process. A PP copolymer powder with 4% ethylene and an ${\;\bar{M}}_{w}$ of $276,000\;g\cdot {\mathrm{mol}}^{-1}$ was graciously supplied by Boréalis (Austria) and was used as a reference hydrophobic matrix.

Particles of 45S5 Bioglass^®^ (Noraker, France) with a chemical composition of (in wt%) SiO_2_, 45; CaO, 24.5; P_2_O_5_, 6; NaO_2_, 24.5, a density of 2.64 g·cm^−3^ and an amorphous microstructure were mixed with PDLA [24]. Different particle sizes (3.5 µm, 40–500 µm) and thermal treatments (hereafter referred as T1, T2 and T3 similar to those carried out in Magallenes et al. [10]) were used in this study. The neat Bioglass^®^ particles were sieved and referenced as BG 3.5 µm NT and BG 0.04–0.5 mm NT, respectively (NT means non-thermally treated BG). Quantitative analysis after thermal treatment was performed using Rietveld quantitative amorphous content analysis (RQACA) [10]. Table 1 describes the effect of the thermal treatments on the studied BG crystallinity.

### 2.2. Biocomposite Preparation: Extrusion-Injection Coupled

PDLA powders were first prepared from the original polymer granules to ensure homogeneous dry blends and were dry mixed with 0, 10, 30 or 50 wt% BG particles of different natures. These Composites are referenced as PDLA(X)BG(Y)(Z), where X, Y and Z represent, respectively, the wt.% of BG, their mean particle size and the thermal treatment.

A co-rotating twin screw extruder (PRISM PTW 16/25D, Thermo Electro Polylab System Rheocord RC400P, Germany) was used to prepare the different composites. A special sealed and heated hopper was used. The process parameters for PDLA/BG and PP/BG were: 150 °C and 180 °C, respectively; a screw rotation speed of 40 rpm; and a flow of argon to limit polymer degradation. Melt extrudates were directly molded with a vertical injection press (Minijet, HAAKE, Karlsruhe, Germany) at 150 °C. In this way, thermo-mechanical degradation due to hydrolytic reaction can be reduced.

### 2.3. Rheological Analysis

Rheological experiments involving the neat polymers and composites were performed with an ARES (TA Instruments, New Castle, USA) strain-controlled rheometer, using a parallel-plate geometry (∅=25 mm). Sample discs were placed between the plates and melted. The investigations were carried out at various temperatures under a continuous nitrogen purge and were performed under dynamic frequency sweeps in the linear viscoelastic regime at angular frequencies ranging from 0.1 to 100 $\mathrm{rad}\cdot s^{-1}$.

### 2.4. Size-Exclusion Chromatography (SEC) Analysis

The average molecular masses, ${\;\bar{M}}_{n}$ and ${\;\bar{M}}_{w},\;$of neat PDLA and of the matrix of composites after processing was determined by size-exclusion chromatography (SEC), using a VARIAN Prostar apparatus (RHEODYNE injector, 100 µL) equipped with PL-Gel G4000 HXL to G1000 HXL columns (50-100000 Å). The detection was carried out by a Shodex RI-101 differential refractometer. Chloroform was used as the eluent (flow rate: 0.5 mL). The running temperature was 45 °C. Prior to the analysis, the composites were solubilized in chloroform and the BG particles were removed by filtration. Calibration was performed using polystyrene standards (Fluka certified DIN).

### 2.5. Fourier Transform Infrared (FTIR) Spectroscopy

Infrared spectroscopy of the neat PDLA and its composites was performed using a Nicolet iS10 spectrometer from Thermo Scientific. The resolution was set to 4 cm^−1^, the wavenumber ranged between 4000 to 450 cm^−1^, and 32 scans were recorded.

### 2.6. Scanning Electron Microscopy (SEM) Analysis

The morphology of each sample was observed using SEM (Thermo Scientific, FEI QUANTA 250 FEG, Hillsboro, OR, USA) to evaluate the particle dispersion of BG. The samples were cryo-fractured in liquid nitrogen. They were placed in adhesive carbon tabs (Agar, Stansted, UK) and observed at a low acceleration voltage (2 kV). Laboratory-developed software for image analysis was used to quantify the inter-particular distances and dispersion quality. The reported values of the layer thickness of C-HA formed onto the surface were the average of five different measurements made at different locations within the same layer.

### 2.7. Mechanical Characterization

#### 2.7.1. Compression Test

Compressive testing was conducted on cylindrical samples (D = 6 mm and L = 11 mm). Measurements were conducted using a universal INSTRON 8502 Servo-Hydraulic Dynamic Testing System (Instron, Norwood, MA, USA) with a crosshead speed of 1 mm/min in accordance with NF EN ISO 604:2002.

#### 2.7.2. Dynamic Mechanical Analysis (DMA)

DMA analysis was conducted on rectangular samples (h = 1.5 mm, b = 12.7 mm and l = 60 mm). Measurements were carried out using a DMA Q800 (TA Instruments, New Castle, PA, USA) in a dual cantilever bending mode with an amplitude of 30 μm at a frequency of 1 Hz. Storage and loss modulus were determined as a function of temperature, from 30 to 100 °C at a heating rate of 1 °C/min.

### 2.8. Bioactivity in Simulated Body Fluid (SBF) in Static Conditions

The bioactivity tests were carried out following the procedure reported in the ISO standard: 23317 ISO [25]. The samples were kept immersed for different periods of time (three and seven days) at 36.5 °C in an orbital shaker set at 25 rpm. During the test, each sample was immersed in the same volume of solution calculated by the Kokubo procedure [26]:

(1) $$V_{s}=\frac{S_{a}}{10}$$

where$\;S_{a\;}$is the apparent surface of the sample (mm^2^).

The SBF solution was replaced every two days to compensate for the decrease in cation concentration during the experiment. The layer of C-HA formed was studied using FTIR spectroscopy and SEM.

## 3. Results

### 3.1. Control and Monitoring of PDLA-BG Processing

**Figure 1:**
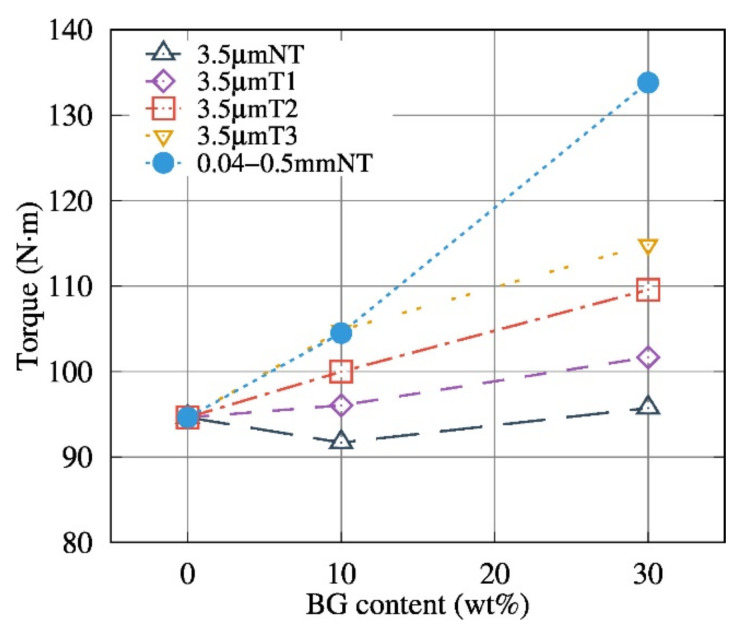
Torque of co-rotating twin screw as a function of BG content during extrusion process at 150 °C with different thermal treatment and particle size as a function of BG content.

Figure 1 shows that the BG particle size has a considerable influence on the melt behavior, of the composites, even at low temperatures (150 °C) and with a low filler content (10 wt.%), with the extrusion process of PDLA with 3.5 µm Bioglass^®^ particle size showing clear signs of degradation (lower torque values than neat PDLA). Process monitoring makes it possible to quantify the PDLA degradation; thermal treatment, particle size and BG content influence the maximum torque measured during the extrusion process.

### 3.2. Morphological Studies

**Figure 2:**
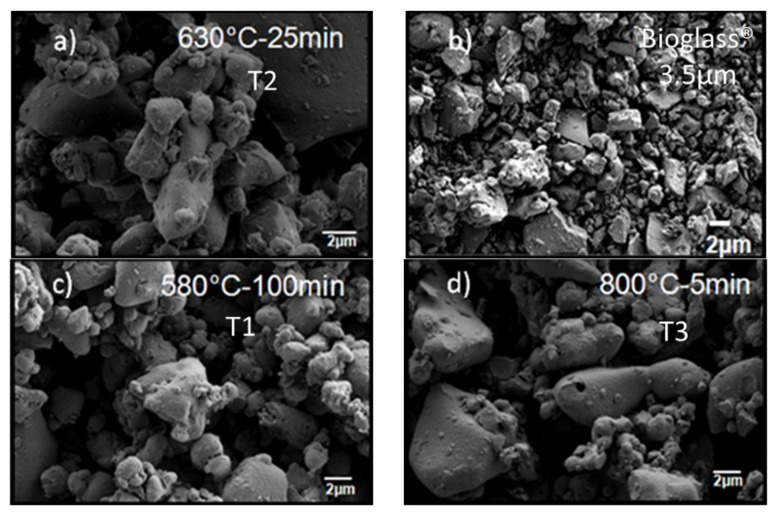
Scanning electron micrograph of the Bioglass^®^ particles, as-ground (**b**) and after thermal treatment (**a**) T2, (**c**) T1 and (**d**) T3.

**Figure 3:**
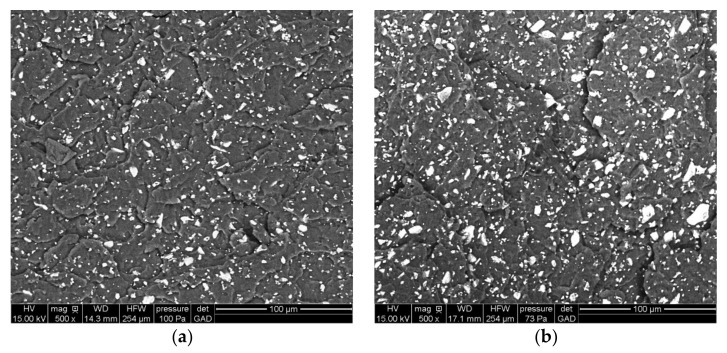
SEM micrographs of cross sections of composite taken in the bulk of (**a**) PDLA30BG 3.5 µm NT; and (**b**) PDLA30BG 3.5 µm T3.

**Figure 4:**
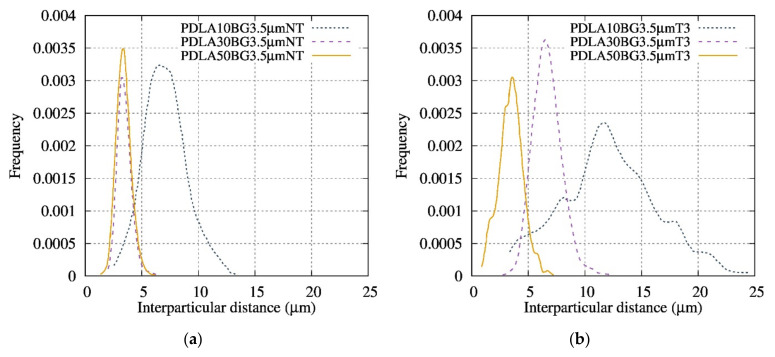
Frequency of inter-particular distances for composites with various contents of (**a**) BG 3.5 µm NT and (**b**) BG 3.5 µm T3.

The morphology of the neat Bioglass^®^ samples was studied by SEM. The roundness of the fillers increases with the temperature of the thermal treatment (Figure 2) and does not influence the particle dispersion in the matrix (Figure 3). The inter-particular distance for BG was obtained by analyzing binary images obtained after segmentation. As shown in Figure 4, the composites exhibited homogenous filler dispersions at different BG contents. The results also reveal that the inter-particular distances logically decreased with increasing BG content and also increased with the thermal treatment for a given fraction of particles. The process developed in this article makes it possible to obtain composite materials with an optimized dispersion.

### 3.3. FTIR Spectroscopy and Degradation Mechanisms

**Figure 5:**
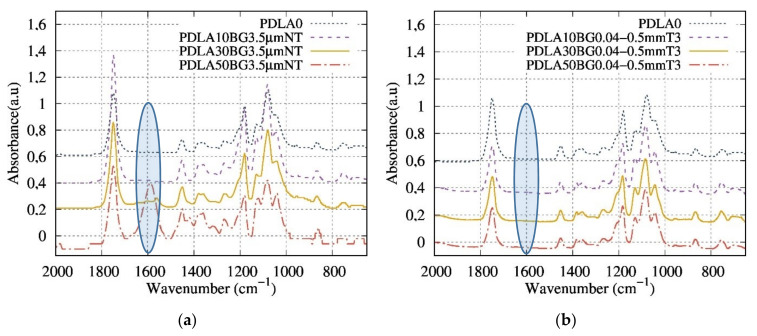
Superposition of (**a**) FTIR spectra of composites containing various contents of BG 3.5 µm NT (wt%); (**b**) FTIR spectra of composites containing various contents of BG 0.04–0.5 mm T3 (wt%).

FTIR spectra of neat PDLA and PDLA/BG composites produced by the extrusion and injection molding process are shown in Figure 5. In the present work, the evolution of the intensity of the absorbance band at 1600 cm^−1^ was monitored as a function of the thermal treatment and BG content of the composites. Blaker et al. [17] and Larrañaga et al. [20], have observed that the FTIR technique highlights the degradation ratio of the composites, with the appearance of an absorbance band at 1600 cm^−1^ corresponding to an increase in PDLA chain-scission. Figure 5a presents a superposition of the absorbance spectra of the composites with various contents of BG 3.5 µm NT. This result shows the presence of the 1600 cm^−1^ band for the composites and its absence for neat PDLA (Figure 5a). In addition, the ratio of the height of the peak, at 1600 cm^−1^ divided by that at 1745 cm^−1^, is the highest with BG 3.5 µm NT, suggesting that the matrix degrades the most significantly when in the presence of these particles.

Figure 5b shows the impact of the BG particle size distribution and confirms the absence of an absorbance peak at 1600 cm^−1^ even with increasing amounts BG 0.04–0.5 mm T3. These results prove that thermally treating the BG and optimizing its size helps to reduce the degradation of the PDLA matrix. Therefore, the combination of T3 and a larger particle size enables a reduction in the PDLA degradation.

The spectra shown in Figure 5 suggest that a chemical reaction occurred between PDLA and BG during the extrusion process leading to the formation of another ester function, according to the following scheme [27]:

(2) $$R-\mathrm{Si}-O¯{\mathrm{Na}}^{+}\;+R\prime -{\mathrm{CO}}_{2}-R\prime \prime \;\to \;R\prime -{\mathrm{CO}}_{2}-\mathrm{Si}-R+R\prime \prime {-O}^{-}{\mathrm{Na}}^{+}$$

### 3.4. Effect of Bioglass^®^ on the Molecular Weight of PDLA

Size-exclusion chromatography was carried out in order to determine the effect of BG filler on the molecular weight of the PDLA matrix. The ratios between ${\;\bar{M}}_{w}$ of PDLA extracted from the composites and ${\;\bar{M}}_{w}$ of the PDLA (${\;\bar{M}}_{w0}$) after the extrusion-injection process were calculated.

**Figure 6:**
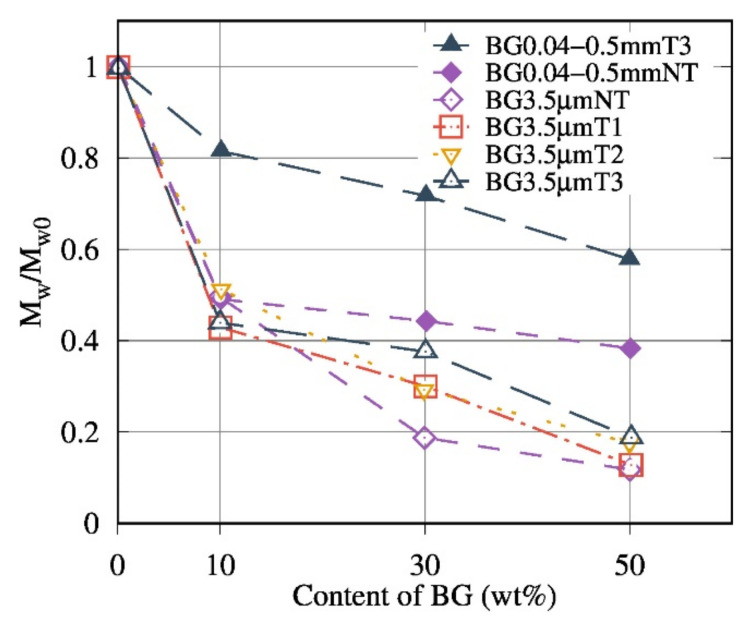
Evolution of the average molecular weight ratios (${\;\bar{M}}_{w}/{\;\bar{M}}_{w0}$) as a function of the BG content for composites with various sizes of BG and with various thermal treatments of BG.

The decrease of the ${\;\bar{M}}_{w}/{\;\bar{M}}_{w0}$ ratio is interpreted as a marker of the degradation of the matrix. Figure 6 shows that ${\;\bar{M}}_{w}/{\;\bar{M}}_{w0}$ decreased with the increase in BG content. The average molecular weight is divided into two zones:

- ≤10 wt% of BG: an especially small decrease of the ${\;\bar{M}}_{w}/{\;\bar{M}}_{w0}$ ratio with the increased BG content was noticeable in the presence of BG 0.04–0.5 mm T3.
- >10 wt% of BG: the ratio of the PDLA50BG 0.04–0.5 mm NT composite stabilized around 0.44 compared to the PDLA50BG3.5μmNT which exhibited a lower ratio (loss of 75%).

Compared to the results obtained by Blaker et al. [17], the degradation rate (${\;\bar{M}}_{w}/{\;\bar{M}}_{w0})$ observed in this work is lower due to the proposed process (Section 2.2).

Composites based on the optimized size and thermal treatment, denoted as BG 0.04–0.5 mm T3, presented the lowest degree of degradation (a loss of 20% for PDLA10BG 0.04–0.5 mm T3). These results also reveal that the addition of BG increased the matrix degradation, especially for smaller BG particle sizes (higher surface contact) with no thermal treatments.

### 3.5. Melt Rheology of Biocomposites

#### Influence of Bioglass^®^ Size and Thermal Treatment

**Figure 7:**
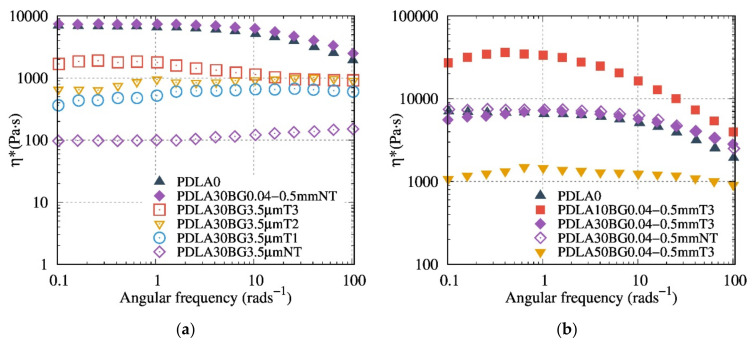
Evolution of the complex dynamic viscosity versus the angular frequency at 150 °C for composites (**a**) with 30 wt% BG as a function of the particle size for NT particles and the thermal treatment of the filler; and (**b**) based on 0, 10, 30 and 50 wt% of 0.04–0.5 mm T3.

**Figure 8:**
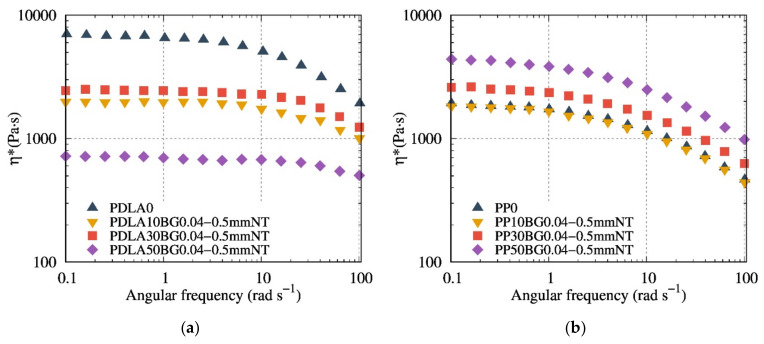
Evolution of the complex dynamic viscosity modulus versus the angular frequency (**a**) at 150 °C for PDLA composites based on 0, 10, 30 and 50 wt% of 0.04–0.5 mm NT in comparison to (**b**) PP composites based on 0, 10, 30 and 50 wt% of 0.04–0.5 mm NT at 180 °C.

**Figure 9:**
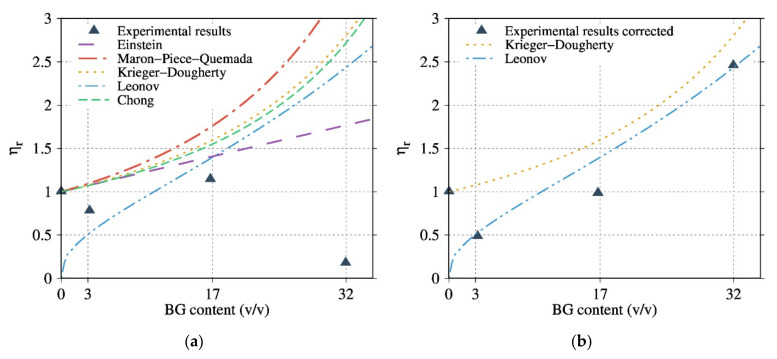
Relative viscosities or hydrodynamic interaction versus the BG volume percentage for composites of PDLA filled with BG 0.04–0.5 mm T3. Comparison between (**a**) the measured experimental values of the relative viscosities and the Einstein, Maron-Piece-Quemada, Krieger-Dougherty, Leonov and Chong et al. models and (**b**) the corrected relative viscosities calculated from the real ${\;\bar{M}}_{w}$(Equations (10) and (11)) values compared with the theoretical relative viscosities calculated from the Leonov and Krieger-Dougherty Leonov models.

The results of the rheological study with controlled frequency sweep of the composite are shown in Figure 7, Figure 8 and Figure 9.

Figure 7a presents the rheological results, highlighting the evolution of the complex dynamic viscosity modulus versus the angular frequency for the composites based on 30 wt% BG with various thermal treatments (T1 to T3). Composites with a thermally treated BG or with a particle size of 0.04–0.5 mm presented a higher viscosity than composites with 3.5 µm NT BG particles. The recorded rheological behaviors reveal that thermal treatments of BG help to avoid the polymer matrix degradation. Thus, T3 is more efficient than T1 and T2 in reducing the PDLA degradation after processing. The rheological measurements demonstrate that the composites with BG 0.04–0.5 mm T3 fillers exhibited the highest viscosity modulus (Figure 7b), combining the influence of TT and of high particle size.

The melt rheological properties of composites based on PDLA and PP, with the BG possessing a particle size of 0.04–0.5 mm at different contents, are shown in Figure 8. The contribution of BG to the viscosity and the hydrodynamic interaction was first proved using a non-degraded matrix (i.e., PP). As compared to PDLA, PP composites demonstrate a classic behavior of composites viscosity, the higher the filler content, the higher the viscosity, regardless of the nature and/or size of the BG.

Compared to PDLA composites with 0.04–0.5 NT (Figure 8a), 0.04–0.5 mm T3 (Figure 7b) composites show a higher viscosity modulus, suggesting that T3 fillers contribute to preventing degradation. PDLA10BG 0.04–0.5 mm T3 (Figure 8b) exhibits a higher complex viscosity modulus than does neat PDLA (which is consistent with the typical behavior of composites), suggesting a lower degradation rate. Nevertheless, PDLA composites with BG 0.04–0.5 mm T3 do not follow the same rheological model as PP (Figure 8b), suggesting a combination of degradation and reinforcement as a result of filler addition.

### 3.6. Modelling the Rheological Behaviour of PDLA/BG Biocomposites

The rheological behavior of PDLA/BG suspension under real experimental conditions has been modeled in order to better understand these properties. As reported in Equation (3), the hydrodynamic interactions, $\psi \left(\phi \right)$, can be calculated from the complex viscosity modulus of the suspension ($\eta^{*}\left(\phi ,\omega \right))$ and the viscosity $\eta^{*}\left(0,\omega \right)$ of the matrix.

(3) $$\eta^{*}\left(\phi ,\omega \right)=\eta^{*}\left(0,\omega \right)\cdot \psi \left(\phi \right)$$

where ω and ϕ are respectively the angular frequency and volumetric fraction of the filler, and $\psi \left(\phi \right)\;$is a function that represents the hydrodynamic interaction.

The rheological behavior was modeled with classical equations, such as those of Einstein (Equation (4)) [28], Maron-Piece-Quemada (Equation (5)) [29], Krieger-Dougherty (Equation (6)) [30], Leonov (Equation (7)) [31] and Chong (Equation (8)) [32]:

(4) $$\psi \left(\phi \right)=\eta_{r}=\frac{\eta_{composite}}{\eta_{matrix}}=1+\left[\eta \right]\phi$$

(5) $$\psi \left(\phi \right)={\left(1-\frac{\phi }{\phi_{m}}\right)}^{-2}$$

(6) $$\psi \left(\phi \right)={\left(1-\frac{\phi }{\phi_{m}}\right)}^{-\phi_{m}\left[\eta \right]}$$

(7) $$\psi \left(\phi \right)=\frac{9\phi^{1/3}}{8\left(1-\phi^{1/3}\right)}$$

(8) $$\psi \left(\phi \right)={\left(1+0.75\frac{\phi }{\phi_{m}\left(1-\frac{\phi }{\phi_{m}}\right)}\right)}^{2}$$

where, [*η*] is an intrinsic viscosity (2.5 for spherical particles), $\eta_{r}(\eta /\eta_{0})$ is the relative viscosity between composite and matrix, $\phi_{m}$ is the maximum particle packing fraction at which the viscous flow can occur and $\psi \left(\phi \right)$ is a function describing the hydrodynamic interactions. The fitting parameters were $\phi_{m}$ = 0.69 and [*η*] = 2.4. These values are valid in a random arrangement of BG particles with a particle size distribution following a Gaussian function.

Assuming that the hydrodynamic interactions dominated at high frequencies (100 rad/s), the dependence of the viscosity could be described by applying the aforementioned models (Equations (4)–(8)).

**Figure 10:**
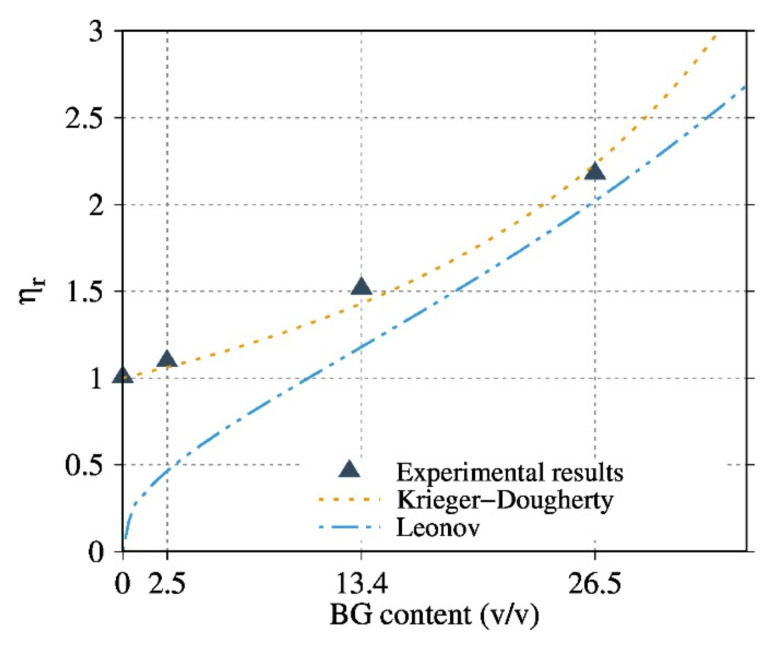
Relative viscosities or hydrodynamic interaction versus the BG volume percentage for composites of PP filled with BG 0.04–0.5 mm T3. Comparison between the experimental values of the relative viscosities calculated from the real ${\;\bar{M}}_{w}$values and the theoretical relative viscosities calculated from the Krieger-Dougherty and Leonov models.

Contrary to PP composites (Figure 10), theoretical models did not describe the viscosity behavior (Figure 9a) since the molar masses change with PDLA degradation. Hence, the real zero-shear viscosity was calculated from SEC measurements using the relationship between the zero-shear viscosity and the weight-average molar mass of a linear polymer, and was subsequently integrated into the corrected relative viscosity (Equation (10))

(9) $$D=\frac{{\left(\eta_{0}\right)}_{corrected}}{\eta_{0}}={\left(\frac{{\bar{M}}_{w\left(real\right)}}{{\bar{M}}_{w\left(0\right)}}\right)}^{\alpha }$$

(10) $$\psi \left(\phi \right)={\left(\frac{\eta_{c}}{\eta_{0}}\right)}_{corrected}=\frac{\eta_{c}}{\eta_{0}}\cdot \frac{1}{D}$$

where *D* is the corrective factor α = 3.4 for ${\;\bar{M}}_{w}{\text{>}M}_{c}$*,* α = 1 for ${\;\bar{M}}_{w}{\;>\;M}_{c}$ ($M_{c}$ is the critical molecular weight of PDLA, $M_{c}=2M_{e}=8900{\;g\text{·}\mathrm{mol}}^{-1}$ and $M_{e}$ is the entanglement molecular weight),$\;{\bar{M}}_{w\left(real\right)}$ and ${\bar{M}}_{w\left(0\right)}\;\mathrm{represent}\;$the weight average of the molar mass of the PDLA of the composite and the neat PDLA respectively.

Figure 9b and compare the experimental results with different models considering the corrected relative viscosity for the PDLA composites. The results are given only for the composites based on the optimized BG 0.04–0.5 mm T3. The Krieger-Dougherty and Leonov models satisfactorily describe the experimental viscosity properties of the PP/BG and PDLA/BG biocomposites, respectively, especially at high BG contents for PDLA. The parameters of the Krieger-Dougherty equation have a physical significance: they consider the effect of polydispersed systems and the particle geometry. It has been observed that, in the case of isotropic suspension and low shear rate conditions, the K-D equation fits well with the experimental results.

The Leonov model considers that the total stress measured is the contribution of the viscoelastic stress from the micro-flow of polymer matrix around the particles, and the stress from particle-particle interactions. This model is more sensitive to the matrix viscosity and better describes the hydrodynamic interaction of PDLA/BG at high content.

Hence, it is possible to quantify and model the hydrodynamic interactions of PDLA/BG.

### 3.7. Mechanical Properties of PDLA/BG Biocomposites

**Figure 11:**
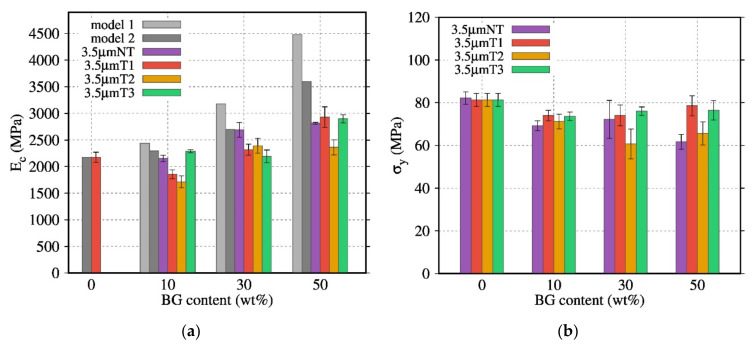
Evolution of (**a**) Ec (MPa), and (**b**) σy(MPa) of composites based on BG 3.5 µm NT with various thermal treatments as a function of the BG content.

The influence of the thermal treatment and content of BG on the compressive mechanical properties was also investigated for composites with BG 3.5 µm and BG 0.04–0.5 mm. The Young’s modulus, obtained by compressive tests at room temperature in the elastic domain, was compared with the mechanical models presented by Takayanagi et al. [33] and Okamoto et al. [34] (Equations (11) and (12)). Figure 11a indicates that above 30 wt% of BG, the modulus Ec of the composites was higher than the Ec of the neat PDLA. Usually, Ec increases when the BG content increases. Furthermore, the biocomposites were subjected to a dynamic mechanical solicitation in order to monitor their response as a function of temperature.

Contrary to the previous results [17], the stresses (σy) were hardly affected by the increase in BG content (Figure 11b). In this study, both yield stresses and modulus (Figure 11a) are sensitive to the PDLA degradation, especially with BG 3.5 µm NT. As a result, the mechanical properties of PDLA at high content of BG particles composites does not follow the proposed model.

(11) $$E_{c1}={\left(\frac{\varphi }{\lambda E_{p}+\left(1+\lambda \right)E_{m}}+\frac{1-\varphi }{E_{m}}\right)}^{-1}$$

(12) $$E_{c2}=\lambda {\left(\frac{\varphi }{E_{p}}+\frac{1-\varphi }{E_{m}}\right)}^{-1}+\left(1-\lambda \right)E_{m}$$

where $E_{p}\left(76\;\mathrm{GPa}\right)$ and $E_{m}(2.15\;\mathrm{GPa})$ are the Young’s modulus of the particles and the matrix respectively, and λ and φ are the parameters depending on the volume fraction of the particles *Vp*, as follows:

(13) $$\lambda =\frac{2+3V_{p}}{5}$$

(14) $$\varphi =\frac{5V_{p}}{2+3V_{p}}$$

Although PDLA30BG 3.5 µm NT presented higher *Ec* values compared to composites with thermally treated BG, it showed high standard deviations and its σy values decreased when the BG content was raised. However, the composites with BG 3.5 µm T3 presented a behavior close to that of neat PDLA. The brittle properties of composites with micrometric fillers had been previously observed [35,36]

**Figure 12:**
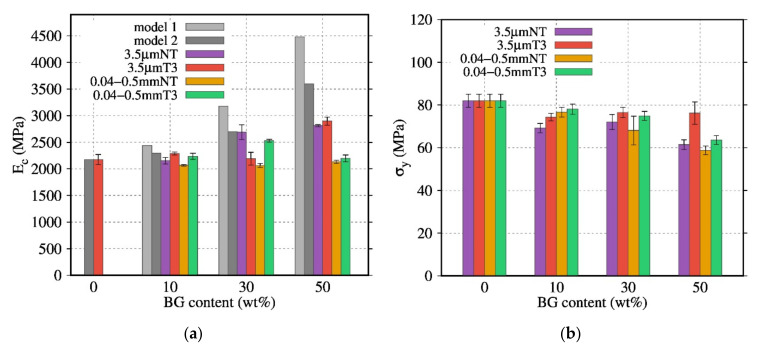
Evolution of (**a**) Ec (MPa), and (**b**) σy (MPa) as a function of the BG content for composites filled with BG of various sizes and thermal treatment.

Similarly, the influence of the BG particle size (BG 3.5 µm NT, BG 0.04–0.5 mm) on the compressive mechanical properties was investigated (Figure 12). The composites with BG 0.04–0.5 mm NT and BG 0.04–0.5 mm T3 displayed a low dependence of the Young’s modulus on the BG content. Their $E_{c}$ values were close to those of neat PDLA with a low standard deviation. Nevertheless, the σy of these composites was affected by the BG content: σy decreased when the BG content was raised.

**Figure 13:**
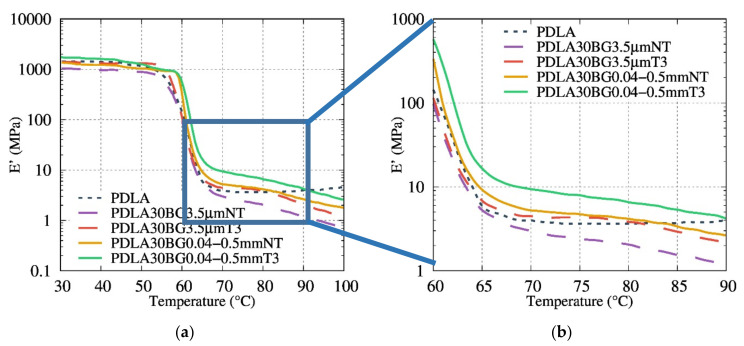
(**a**) Evolution of E’ (MPa) as a function of the temperature for composites filled with BG of various sizes and thermal treatment; (**b**) zoom of rubber plateau modulus.

Even for biocomposites with a high degradation rate, the Ec and σy are close to the values observed for the biocomposites with a less degraded matrix (biocomposites with the thermal treatment T3 and the particle size 0.04–0.5 mm), suggesting that the interface between the BG particles and PDLA matrix is in the origin of the fracture. Furthermore, composites with T1 and T3 exhibit a higher Young’ modulus and yield stress. However, compared to rheological analysis, the use of bigger particles did not show a positive effect on the yield stress; DMA analysis in Figure 13 shows the effect of heat treatment and particle size on the mechanical properties. The filled PDLA30BG 0.04–0.5 mm T3 display the higher plateau modulus in comparison with the non-treated ones. The loss on the rubber plateau modulus, in comparison with the neat PLA, highlights the signature of PDLA degradation on the presence on non-optimal BG.

### 3.8. In Vitro Bioactivity Studies

**Figure 14:**
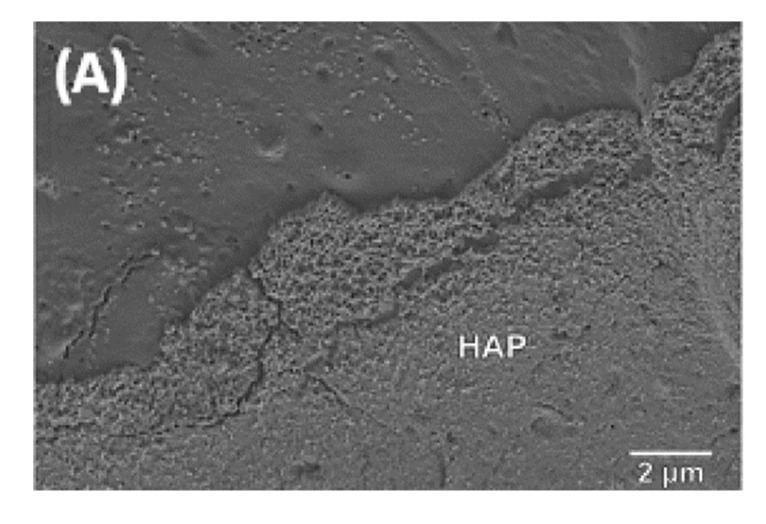

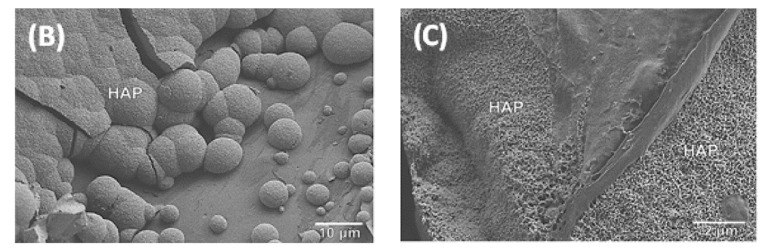
SEM images of biocomposites: (**A**) PDLA10BG 3.5 µm T3 after three days in SBF; (**B**) PDLA50BG 0.04–0.5 mm NT after seven days in SBF; (**C**) PDLA50BG 0.04–0.5 mm T3 after 7 days in SBF.

Samples with different BG particle sizes, content and thermal treatments were subjected to in vitro testing in order to estimate the ability of HA formation in a model-simulated body fluid. The analysis of HA formation on the surface of the composite in SBF can be used as a tool to predict the in-vivo bioactivity [26]. As shown in Figure 14, after seven days in SBF, a layer covering the surface was detected by SEM.

Figure 14 shows the cauliflower-like structure typical of the formation of C-HA on top of the biocomposites surface. Table 2 confirms that a small particle size, low crystallization and an increase of particle BG content enhances the kinetics of bioactivity of the composites. The specific area of BG particles is higher for small particles: an increase in the interface between BG and PDLA enables faster dissolution of BG and enhances bioactivity. The crystallinity of BG, induced by the thermal treatment, produces less soluble BG which decreases the kinetics of bioactivity.

All of the composites with a 50 wt% content of BG subjected to in-vitro testing developed an HA layer after seven days in SBF.

## 4. Discussion

In this work, PDLA degradation was first monitored during its melt extrusion with BG fillers. The process monitoring (temperature, time and torque) allowed for the defining of a processing window to limit this degradation. The use of a special twin screw profile, the use of inert gas and the development of an in-situ and one step hybrid extrusion-injection process limited the PDLA degradation. As a result, PDLA/BG composites with optimal thermal treatments and bigger particle sizes showed limited coloration, a smooth surface and high torque values.

Secondly, the present degradation mechanism was investigated by FTIR measurements and the molar mass of the PDLA after processing was quantified by SEC.

Previous works have studied the variations between the peaks of neat PDLA and composites [17,18] in FTIR. Blaker et al. [17] reported a possible reaction between PDLA and BG during the extrusion process with the counterions Na^+^ or Ca^2+^ as follows:

(15) $${R-\mathrm{Si}-O}^{-}{+R\prime -\mathrm{CO}}_{2}-R\prime \prime \;\to \;R-\mathrm{Si}-O-R\prime \prime {+R\prime -\mathrm{CO}}_{2}^{-}$$

In our study, degradation was detected by the appearance of an absorbance band at 1600 cm^−1^ and a reduction in intensity of the peak at 1750 cm^−1^ in composite samples. These bands can be attributed to the stretching band of the carbonyl function of a new ester group and to the carbonyl function of the ester group of poly(α-hydroxyesters) respectively.

The first reaction mechanism (Equation (15)) seems not explain the R-Si-O- anion attacks and couples with the R” group, rather than the electrophilic carbon of the ester function. Based on this study, another possible reaction mechanism, which seems to be more realistic and closer to the trans-esterification reaction, was proposed in this study (Equation (2)).

The SEC study, then, confirmed the obtained results by FTIR. Hence, we can assume that optimal composites with at least no presence of an absorbance band at 1600 cm^−1^ have the highest Mw/Mw0 value and, consequently, lower degradation.

Our finding highlights that a coupling between the optimal BG size and their thermal treatment could be a way to control the PDLA degradation during the preparation of its biocomposites. Indeed, the obtained SEC results corroborate FTIR measurements and torque monitoring. Composites based on BG 0.04–0.5 mm T3 exhibit the lowest matrix degradation (since there is no signature of degradation recorded by FTIR measurements). Furthermore, melt shear rheology seems to be a suitable tool to probe the PDLA degradation and effects of BG filler content and nature. In contrast to BG 3.5 µm NT based composites, suspensions based on BG 0.04–0.5 mm T3 presented the highest complex dynamic viscosity, even at high content of BG. Interestingly, we have quantified the hydrodynamic interactions, comparing different suspensions models and taking into account the real PDLA viscosity value during rheological measurements. Considering the matrix degradation quantified by SEC, it was possible to quantify and model the real hydrodynamic interaction of PDLA/BG composites using Leonov equation.

Thermomechanical properties of the studied composites were investigated. Special attention was paid to how BG fillers influence the viscoelastic properties above the glass transition. The composites with a higher degradation amount, PDLA30BG 3.5 µm NT and PDLA30BG 3.5 µm NT, presented a decrease and loss in the length of the plateau region, which indicates the lower molar masses of the degraded PLA. However, the use of thermal treated and larger particles showed a positive effect on the thermal resistance of the storage modulus.

As observed by RQACA, thermal treatments influenced the crystallinity of BG particles. The bioactivity test in SBF under static conditions showed that the specific surface and crystallinity of BG had an influence on the bioactivity. Biocomposites with high specific surface (3.5 µm) and amorphous (NT) BG accelerated the formation of the HA layer. In particular, 3.5 µm NT biocomposites developed an HA layer after three days of immersion. On the other hand, biocomposites with a higher particle size and with a thermal treatment presented an HA layer after seven days immersed in SBF. As a result, the crystallinity, the specific surface and the content of BG can be used as a tool to control bioactivity and the matrix degradation.

## 5. Conclusions

This study investigates the thermal degradation of PDLA-based composites during processing for different volume fractions, particle size distribution and phase composition of bioactive glass fillers. PDLA/BG were processed by high temperature extrusion (i.e., melt processing). Through this work, an optimized high temperature extrusion-injection process under argon flow has been developed. The addition of non-thermally treated BG was shown to degrade the PDLA matrix, leading to a reduction in molar masses, viscoelastic and mechanical properties. Results confirmed that PDLA degradation during the extrusion-injection process was principally due to a chemical reaction in the BG/PDLA interface. The great efficiency of thermal treatment T3, especially with BG 0.04–0.5 mm, to limit matrix degradation was shown.

After correcting the relative viscosity by considering the measured molar masses, the model of Krieger-Dougherty satisfactorily described the hydrodynamic interactions. The mechanical analysis (compression test and DMA) corroborated the rheological and physicochemical results. The DMA results demonstrated a higher rubber plateau modulus for the optimal BG fillers.

All bioglasses showed a thin layer of apatite on the sample surface after seven days of immersion in SBF under static conditions. SEM analyses confirmed the formation of C-HA and proved that PDLA/BG composites are bioactive.

In order to control the degradation of biocomposites in-vivo, the present work has demonstrated that by (i) controlling the geometrical, crystalline structure of BG and (ii) optimizing the processing parameters during melt blending, it was possible to limit the PDLA degradation and increase the rheological, physicochemical and morphological properties of these bioactive composites.

## Figures and Tables

**Table 1:** Thermal treatments used in this work, with the resulting crystallinity index.

Name	Temperature (°C)	Time (Minutes)	Crystallinity (%)
T1	630	25	80 ^1^
T2	580	100	10 ^1^
T3	800	5	91 ^1^ and 1 ^2^

^1^ crystals of Na_2_CaSi_2_O_6_, ^2^ crystals of Na_2_Ca_4_P_2_SiO_12_.

**Table 2:** Results obtained by SEM for biocomposites with different BG particle size, content, thermal treatment and immersion time in SBF. With F, formation and NF, non-formation of HA layer.

Particle	wt%	3 Days	7 Days
3.5 µm NT	10	F	F
	30	F	F
	50	F	F
3.5 µm T3	10	F	F
	30	F	F
	50	F	F
0.04–0.5 mm NT	10	NF	NF
	30	F	F
	50	F	F
0.04–0.5 mm T3	10	NF	NF
	30	NF	NF
	50	F	F

## Data Availability

Corresponding authors.
